# Associated Factors for Long-Term Sick Leave or Reduced Work Capacity Due to Musculoskeletal Disorders Among Nursing Staff: An Exploratory Study

**DOI:** 10.3390/healthcare14213715

**Published:** 2026-11-02

**Authors:** Michaela Reimann, Albert Nienhaus, Claudia Westermann, Anja Schablon, Peter Koch

**Affiliations:** 1 Competence Center for Epidemiology and Health Services Research for Healthcare Professionals (CVcare), Institute for Health Services Research in Dermatology and Nursing (IVDP), University Medical Center Hamburg-Eppendorf (UKE), Martinistrasse 52, 20246 Hamburg, Germany; 2 Institution for Statutory Accident Insurance and Prevention in the Health and Welfare Services (BGW), Department of Occupational Medicine, Hazardous Substances and Public Health (AGG), Pappelallee 33-37, 22089 Hamburg, Germany; albert.nienhaus@bgw-online.de (A.N.);

**Keywords:** nurse-work Instability scale, associated factors, musculoskeletal disorders, ergonomic knowledge, use of ergonomics, nursing staff

## Abstract

**Highlights:**

**What are the main findings?**

• Over one-third of respondents are at high risk for Work Instability and therefore at an increased risk of long-term sick leave or reduced work capacity due to musculoskeletal disorders.
• Older age of the nurses, shift work, lack of assistive equipment and moderate-to-poor ergonomic practices were identified as influencing factors.

**What are the implications of the main findings?**

• Having sufficient assistive devices and following good ergonomic practices can reduce the physical strain on nurses.
• A combination of environmental and behavioral prevention measures is necessary to support nurses’ long-term health and work ability.

**Abstract:**

**Background/Objectives:** Musculoskeletal disorders (MSDs) are often the cause of long-term sick leave (LTSL) or reduced work capacity (RWC) among nurses. We therefore investigated the risk of LTSL or RWC due to MSDs among nurses, as well as possible ergonomics-related influencing factors. **Methods:** The cross-sectional study was conducted in 2025 using an online questionnaire. The study population consisted of nursing staff insured with the German Institution for Statutory Accident Insurance and Prevention in the Health and Welfare Services (BGW). The risk of LTSL or RWC due to MSDs was assessed using the German version of the Nurse—Work Instability Scale (Nurse-WIS). In addition, data relating to sociodemographic factors, health behaviour and workplace ergonomics were collected. The exploratory statistical analysis was descriptive, bivariate and multivariate. **Results:** A total of 501 nurses took part in the survey (mean age 45.5 ± 13.4 years; 86.2% female). A total of 36% of participants were identified as being at high risk of LTSL or RWC due to MSDs. The following associated factors were identified for individuals at high risk: age group 50 to 59 (OR 2.8; *p* = 0.001), shift work (OR 1.6; *p* = 0.026), insufficient availability of ergonomic aids (OR 1.8; *p* = 0.004) and a subjective assessment of ergonomic working practices as moderate (OR 2.3; *p* < 0.001) or poor (OR 2.5; *p* = 0.033). **Conclusions:** The proportion of individuals at increased risk of LTSL or RWC due to MSDs was high in the study sample. To protect nursing staff at an early stage, healthcare facilities should ensure the availability of sufficient ergonomic aids and provide regular ergonomic training for staff.

## 1. Introduction

Musculoskeletal disorders (MSDs) are one of the most common health problems in Europe, affecting millions of people in the workforce. Approximately 25% of European workers suffer from back pain and 23% from muscle pain [1]. Compared to other occupational groups, workers in the healthcare professions are particularly prone to MSDs [2], with nursing staff facing a substantial risk [3]. The causes of MSDs include heavy lifting, performing repetitive tasks, maintaining poor postures for extended periods, and working despite fatigue or pre-existing injuries [4,5]. Additional risk factors include psychosocial stress and effort-reward imbalances [6]. This leads to reduced work ability among nursing staff, which continues to deteriorate with age [7]. The consequences include impaired health and quality of life for those affected, as well as a decline in the quality of care for patients [8].

Ergonomics focuses on how tasks can be adapted to a person’s physical and mental resources without limiting their performance or well-being [9]. Teaching ergonomic principles is an integral part of nursing education programs to prevent the development of MSDs [10]. However, a recent review shows that the effectiveness of curricular interventions on ergonomics in nursing education is low [11]. In daily practice, there are difficulties in applying ergonomic knowledge to the daily work of nursing staff [12]. The failure to apply ergonomic principles leads to physical complaints, reduced job satisfaction, and absenteeism among nurses. This results in increased strain on nursing staff due to overtime, further staffing shortages, and unnecessary costs for the healthcare system [7]. These MSD-related periods of incapacity for work, reduction in working hours, and workforce withdrawal place an additional burden on a healthcare system that is already suffering from a shortage of healthcare workers [13].

Incapacity for work is often preceded by a phase of work instability (WI). WI means that, in the period leading up to the actual incapacity for work, those affected are already experiencing increasing difficulty in coping with the demands of their daily work [14]. It has been shown that interventions implemented during the WI phase can prevent the loss of work capacity. Therefore, early identification of affected individuals is important [15,16,17].

The Nurse—Work Instability Scale (Nurse-WIS) is a profession-specific instrument that appears capable of identifying nurses at risk for WI and therefore at increased risk for long-term sick leave (LTSL) or reduced work capacity (RWC) due to MSDs [14]. The predictive ability of the German version of the Nurse-WIS with regard to LTSL or RWC due to MSDs has so far been examined in two German longitudinal studies. These studies revealed moderate levels of specificity (76.7 and 63.4, respectively) and sensitivity (73.9 and 64.1, respectively) [18,19]. Given the predictive power of the Nurse-WIS, this questionnaire can be used in companies as a screening tool to identify employees at risk of LTSL or RWC due to MSDs. It has been found that the risk of premature retirement increases with a history of previous periods of long-term sick leave [20]. The Nurse-WIS appears to be suitable for identifying individuals at risk of LTSL or RWC due to MSDs at an early stage and for initiating timely interventions. This could potentially prevent sick leave and early retirement, which would benefit not only those affected, but also patients and the entire healthcare system. Risk factors for MSD-related incapacity for work among nursing staff have been studied repeatedly [2,21,22,23]. However, job-related risk factors, especially ergonomics-related factors, for the risk of LTSL or RWC due to MSDs among nursing staff have not yet been investigated.

Therefore, this exploratory study aims to answer the following questions:

1. What percentage of nursing staff are at increased risk of LTSL or RWC due to MSDs?
2. Which ergonomics-related factors are associated with an increased risk of LTSL or RWC due to MSDs?

## 2. Materials and Methods

The study was conducted as a cross-sectional survey using an online questionnaire developed specifically for this research. The data collection period was from May to September 2025.

### 2.1. Study Population

The target group for the survey consisted of nursing staff and nursing trainees working in hospital and outpatient care settings. From a total of 21,000 insurance claims related to uncomplicated commuting accidents reported to the German Institution for Statutory Accident Insurance and Prevention in the Health and Welfare Services (BGW) in 2023 and 2024, 4000 nursing professionals were randomly selected and invited to participate in the online survey (a random sample of approximately 20%). Uncomplicated commuting accidents that are not subject to mandatory reporting occur predominantly by chance and are unlikely to be associated with musculoskeletal disorders before or after the accident. These insurance claims are therefore likely representative of the total number of nursing staff insured by the BGW. A commuting accident is not considered reportable if it results in no sick leave or sick leave not exceeding 3 days. Statutory accident insurers in Germany are only aware of their insured individuals once they report a work-related or commuting accident, or a suspected occupational disease. Therefore, it was not possible to randomly select the study population from the total group of all nursing staff insured by the BGW.

Insured individuals who met the inclusion criteria were invited by letter by the BGW’s liaison office to participate in the study. After excluding 643 individuals due to incorrect addresses, employment in occupational groups outside the target population, or having reached retirement age, a total sample of 3357 (83.9%) individuals remained. In the invitation letter, the recipients received the following information about the study: the purpose and procedure of the survey, the voluntary nature of participation, information on data protection, and the anonymous handling of their data without disclosure to third parties. Approval for the study was granted by the “Local Psychological Ethics Committee at the Center for Psychosocial Medicine” (LPEK) of the University Medical Center Hamburg-Eppendorf (UKE) (LPEK-0903).

To participate in the study, potential participants accessed the study website hosted on the UKE’s servers using an individual study ID via a QR code or link provided in the invitation letter. Before the survey began, participants actively gave their consent to participate based on the participation information and privacy policy. This consent could be withdrawn at any time during the survey without providing a reason. At the UKE, data collection and analysis were conducted in a pseudonymized manner, preventing the study ID from being linked to the BGW’s personal data by the participants. After four weeks, the BGW’s data protection office used the individual study IDs to identify individuals who had not yet participated in the survey at that time. The BGW’s data protection office sent these individuals a reminder letter inviting them to participate in the study.

### 2.2. Questionnaire

Sociodemographic, health-related, occupational, and ergonomics-related data were collected. To assess the participants’ educational qualifications, they were asked about their high school diploma and professional qualifications.

Data on the risk of LTSL or RWC due to MSDs were collected using the validated German version of the Nurse-WIS [24]. The questionnaire contains 30 statements that respondents are asked to rate as “Agree” or “Disagree.” A total score (0–30) is calculated from the responses, which is then classified into one of three risk categories: low (<10 points), moderate (10–19 points), or high (≥20 points). Individuals with a score of 20 or higher on the Nurse-WIS are at risk of LTSL or RWC due to MSDs. The Nurse-WIS has proven to be a reliable prognostic tool for predicting the risk of LTSL or RWC due to MSDs [18].

We developed new questions regarding ergonomic knowledge specifically for this survey. To this end, five multiple-choice questions on various aspects of ergonomics were asked (Appendix A). A total score was derived from the correct answers (0 = no knowledge, up to 10 = extensive knowledge). The score was dichotomized based on the median (scores below 8 points: low to medium knowledge; scores of 8 points or higher: high knowledge). To conduct a pretest regarding the difficulty level and comprehensibility of the questions, a member of the nursing team distributed the questionnaire to 20 licensed healthcare and nursing professionals at a university hospital. Using questions developed specifically for the survey, data were collected on participation in ergonomic training courses over the previous two years, the availability of ergonomic aids in the facilities, and their use by the participants. Participants’ subjective assessment of their own ergonomic work practices was also measured using a question developed specifically for the study: “Overall, how would you rate your own ergonomic work practices?” Participants rated their own ergonomic work practices on a six-point scale ranging from 1 (very good) to 6 (unsatisfactory). The newly developed questions have not undergone prior validation.

### 2.3. Outcome Measures

The outcome measure was the proportion of nurses who, according to the Nurse-WIS, were at high risk for LTSL due to MSDs. Independent variables included sociodemographic, health-related, occupational, and ergonomics-related data.

### 2.4. Statistical Analysis

Categorical variables were presented as frequencies and proportions; continuous variables were presented as means with standard deviations. For further statistical tests, the variables “occupational group” and “setting” were combined. To investigate associations between dependent and independent variables, odds ratios (OR) with corresponding 95% confidence intervals (CI) were first calculated using univariate logistic regression analyses. From the univariate analyses, all variables with a *p*-value <0.25 were included in the baseline model for the multivariate logistic regression analysis [25]. Using the backward selection method, the multivariate models were then reduced to significant predictors [26]. The remaining covariates were checked for interaction and multicollinearity and, where confirmed, only one of the affected variables was included.

To investigate possible nonresponse bias, an analysis of nonrespondents was conducted using the random sample. The influence of age, gender, occupation, and setting on the response rate was examined. Data from the routine dataset were used for the nonresponder analysis. Significance tests (chi-square tests) were used to examine systematic differences. Data collection was conducted using the Unipark online platform, and subsequent data analysis was performed using the Statistical Package for the Social Sciences (SPSS), Version 29 (IBM). The significance level was set at *p* < 0.05.

## 3. Results

The response rate relative to the number of people originally contacted was 15.6%. Of the 523 data sets originally included, all cases with missing values for the following variables were excluded prior to analysis: age, gender, Nurse-WIS, assistive technology use, and subjective assessment of one’s own ergonomic work practices. After cleaning the data, 501 data sets were included in the analysis.

### 3.1. Sample Description

86.2% of the participants were female, and their mean age was 45.5 (SD: 13.4) years (Table 1). The average weekly working hours were 34.7 (SD: 9.9) hours, and the respondents had been employed in nursing for an average of 20.8 (SD: 13.4) years. Certified health and nursing professionals accounted for the majority of respondents at 59.8%, while 9.5% were certified geriatric care workers and 18.6% of the participants reported having a qualification as a nursing assistant. 52.9% reported having a secondary school diploma as their highest level of education. Slightly more than half of the participants (57.6%) worked in a hospital, 64% worked shifts, and 19.5% held a management position. Nearly half of the respondents (48.5%) were employed full-time. Just over 60% of the participants reported engaging in physical activity for less than two hours per week, and 25.4% were smokers. 34.5% were of normal weight, 1.2% were underweight, and a total of 61.2% fell into the overweight and obese Body Mass Index (BMI) categories. Two-thirds of the respondents had attended an ergonomic training course within the past two years. More than half of the respondents (56.5%) consider the supply of ergonomic aids at their respective facilities to be insufficient. When asked about their own use of available aids, 73.2% reported frequent use, 21.9% moderate use, and 5.0% rare or no use of these aids. The majority of respondents (67.1%) possessed a high level of ergonomic knowledge. Slightly less than half of the participants rated their own ergonomic work practices as good (47.7%) or moderate (46.9%). Only 5.4% of respondents rated their own ergonomic work practices as poor.

### 3.2. Risk of LTSL or RWC Due to MSDs

**Figure 1:**
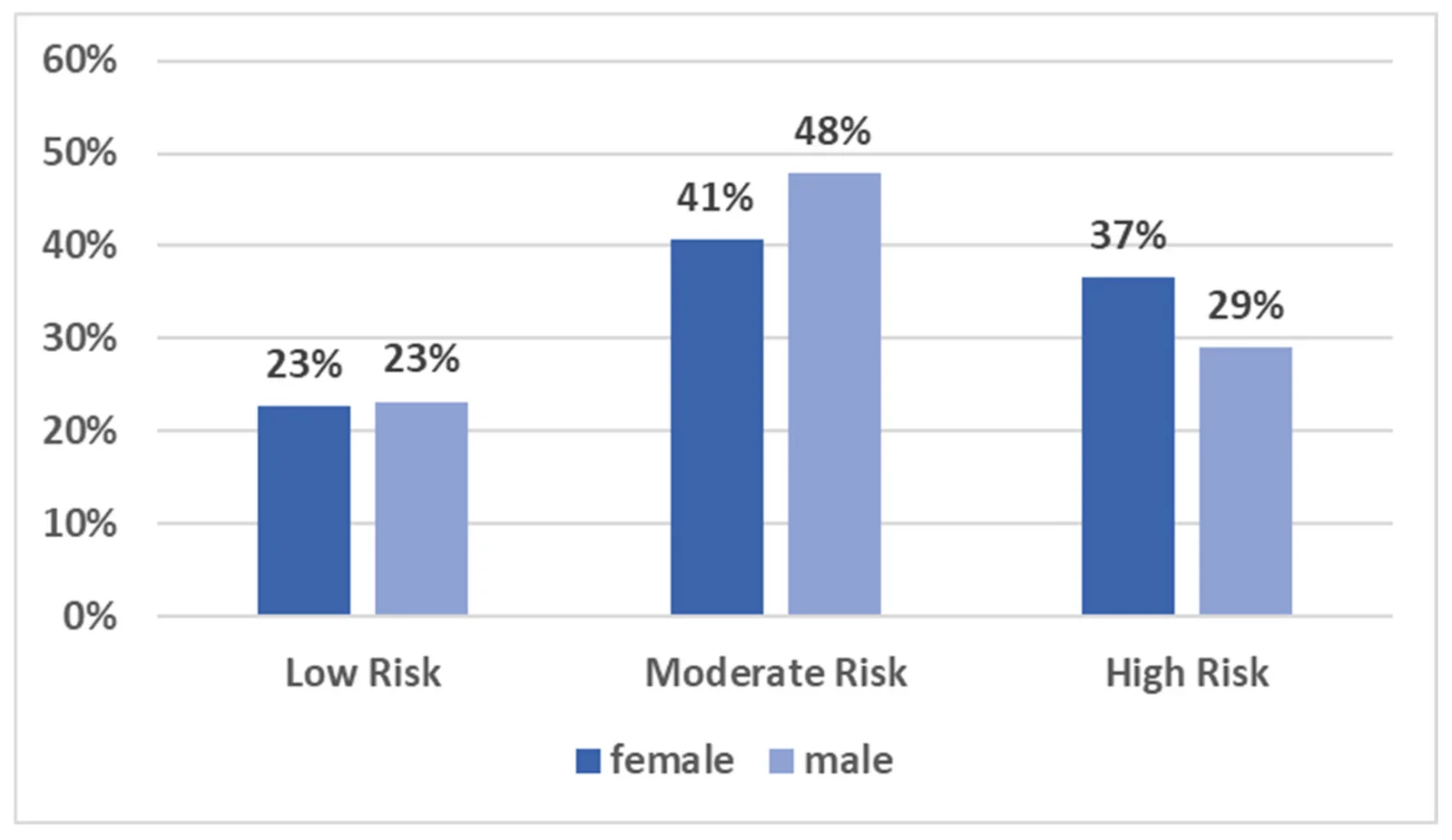
Distribution across the Nurse-WIS risk categories by gender (*n* = 501).

According to the Nurse-WIS, 22.8% of the overall group had a low risk, 41.7% had a moderate risk, and 35.5% of respondents had a high risk of MSD-related LTSL or RWC. No statistically significant differences were observed between women and men (*p* = 0.430) (Figure 1).

In the bivariate analysis, the availability of ergonomic aids in the facilities and the subjective assessment of one’s own ergonomic work practices were found to have a statistically significant influence on classification into the highest risk category (Table 2).

Individuals who rated the assistive equipment resources at their current facility as insufficient had an increased OR of 1.9 (95% CI = 1.34–2.88) compared to individuals from facilities with sufficient resources. Increased ORs were also observed for nursing staff who rated their own ergonomic work practices as moderate or poor (OR 2.1; 95% CI = 1.43–3.10 and OR 3.0; 95% CI = 1.34–6.75, respectively). Women (OR 1.4; 95% CI = 0.81–2.46), individuals working shifts (OR 1.5; 95% CI = 0.99–2.16), smokers (OR 1.5; 95% CI = 0.98–2.24), and participants who engaged in physical activity for less than two hours per week (OR 1.4; 95% CI = 0.95–2.24) also had increased ORs compared to the respective reference group. The age of the respondents also influenced their classification into the highest-risk category of the Nurse-WIS. Here, nurses between 50 and 59 years showed the highest increase in risk, with an OR of 1.8 (95% CI = 1.00–3.14). However, the associations between the last five factors and the outcome were not statistically significant.

**Figure 2:**
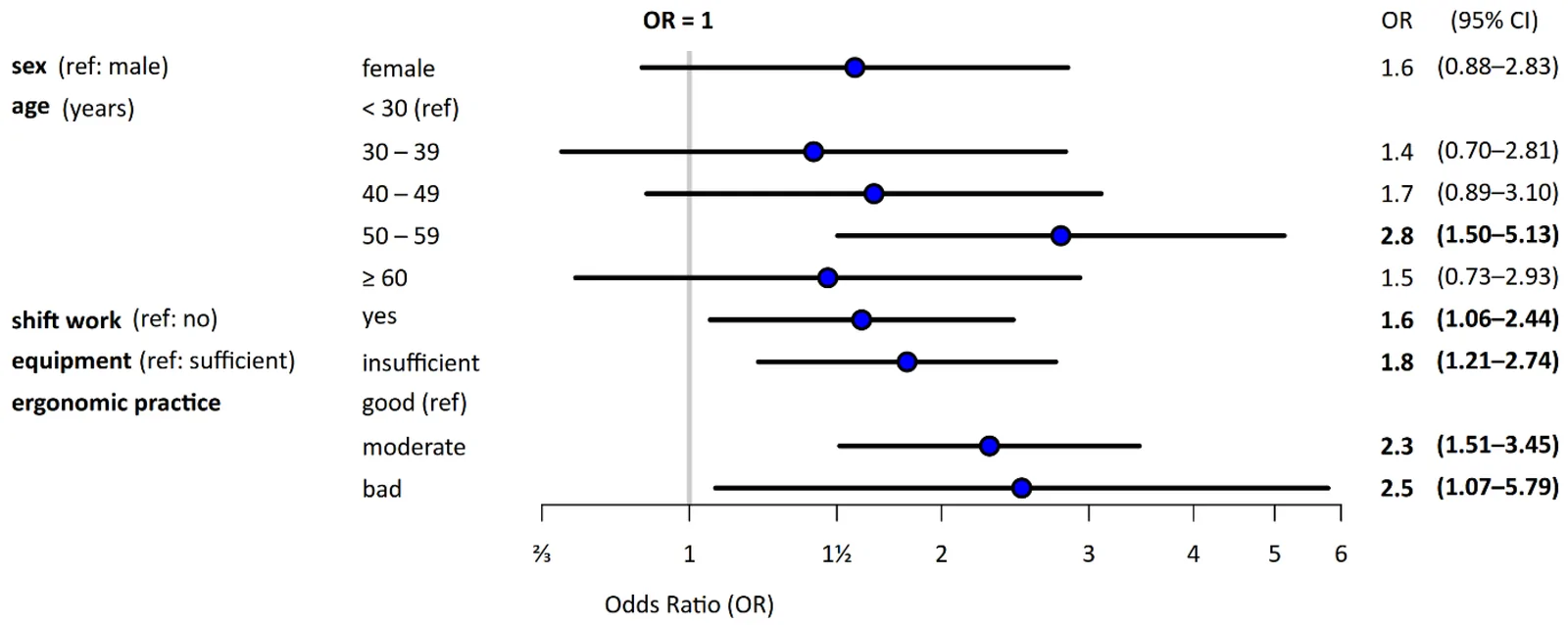
Predictors of a high risk of LTSL or RWC due to MSDs (*n* = 501) multivariate logistic regression; ref: reference group; 95% CI: 95% Confidence interval; **bold:** significant (*p* < 0.05).

In the multivariate model, shift work, the availability of assistive devices at the workplace, the subjective assessment of one’s own ergonomic work practices, and age also had a statistically significant influence on the probability of a high risk of LTSL or RWC due to MSDs (Figure 2).

Compared to individuals who rated their own ergonomic work practices as good, those who rated their own work practices as moderate or poor had elevated ORs (OR 2.3; 95% CI = 1.51–3.45; *p* < 0.001 and OR 2.5; 95% CI = 1.08–5.79; *p* = 0.033). Individuals working in facilities without adequate assistive devices had an OR of 1.8 (1.21–2.74; *p* < 0.001) compared to the reference group. The probability of falling into the high-risk category was also elevated for nursing staff working shifts compared to those who did not work shifts (OR 1.6; 95% CI = 1.06–2.44; *p* = 0.026). Age also represented a statistically significant associated factor: For individuals in the 50–59 age group, an OR of 2.8 (95% CI = 1.50–5.13; *p* = 0.001) was observed compared to the reference group.

### 3.3. Results of the Non-Responder Analysis

The results of the non-responder analysis are presented in Table 3. Statistically significant differences in response rates were observed with respect to gender, age, occupational group, and setting. Men participated in the survey less frequently than women (10.1% versus 14.4%, *p* = 0.002). The lowest response rate was among insured individuals under 30 years of age (7.8%). Certified nursing staff participated more frequently than nursing assistants (14.6% versus 8.6%, *p* < 0.001). Hospital employees participated more frequently than those working in outpatient settings (15.6% versus 11.8%, *p* < 0.001).

## 4. Discussion

The risk of LTSL or RWC due to MSDs, as well as associated factors, was analyzed in a sample of 501 nursing staff from outpatient and inpatient settings. In our study, 35.5% of respondents showed a high risk of LTSL or RWC due to MSDs according to the Nurse-WIS. No statistically significant differences were found between women and men. Shift workers, facilities with inadequate ergonomic equipment, individuals with moderate-to-poor ergonomic work practices, and older employees were identified as risk groups for LTSL or RWC due to MSDs. Contrary to expectations, the level of knowledge regarding ergonomic work practices had no significant influence on the outcome.

Our data confirm the findings of an earlier study from Germany, in which the proportion of nursing staff at increased risk for LTSL or RWC due to MSDs was 39.4%—only slightly higher than the 35.5% found in the current study [19]. In a study from Brazil, the proportion of respondents at high risk according to the Nurse-WIS was 30.6%, which is lower than in our study. It should be noted, however, that the Brazilian-Portuguese version of the Nurse-WIS has fewer items, so the comparability of the results is limited [27]. Compared to the results of Harling et al., our findings are significantly higher than the 23.7% [24] and 23.3% [18] observed in the high-risk group. In contrast to the present study, these two studies surveyed exclusively employees in long-term care for the elderly, which could explain the difference in the results. In our study, no statistically significant differences were observed between women and men. Thus, our results confirm those of an earlier study [27].

### 4.1. Age as Associated Factor

To date, the literature has not examined factors associated with the Nurse-WIS. For this reason, the following comparisons cite studies that have examined factors associated with absence from work due to MSDs or illness. Our survey showed that the likelihood of a high risk of LTSL or RWC due to MSDs increases with age. Our study thus confirms the findings of two recent reviews that identified age as a significant risk factor for absence from work due to illness [28,29]. One reason for this is the increased prevalence of chronic conditions such as MSDs with advancing age [30,31]. This increased susceptibility can be explained by the loss of physical strength and reaction time that accompanies aging. This can lead to limitations in daily work life, such that affected individuals can no longer meet the demands of their jobs and their work ability declines [32]. Another factor that increases the risk of absence from work due to MSDs among older workers is psychosocial demands in the workplace as well as work-time-related exposures [29]. This increased vulnerability to chronic diseases is even more pronounced in physically demanding occupations among older workers. Heavy lifting (loads of 15 kg or more), pulling or pushing heavy loads, repetitive movements, and working with the upper body twisted or bent forward have an even greater impact on the risk of long-term sick leave among older workers compared to their younger colleagues [29,31]. This effect can also be observed in physically demanding care professions, where MSDs are common across all age groups. Garzaro et al. [7] found a particularly marked increase in the prevalence of MSDs and, consequently, a decline in work ability among those aged 46 to 55 as well as among employees aged 56 and older. These findings were confirmed by our study, in which the group of respondents aged 50 to 59 showed the highest risk of LTSL or RWC due to MSDs. However, the results of our study show a decrease in the risk of LTSL or RWC due to MSDs in the age group ≥60 years, which contradicts the findings of the study by Garzaro et al. [7]. One possible explanation for the reduced OR in this age group is the healthy worker effect.

### 4.2. Shift Work as Associated Factor

Our study identified shift workers as another group at increased risk for LTSL or RWC due to MSDs. Our findings thus confirm the results of earlier studies [33,34]. Previous publications have shown that this effect is further amplified with increasing age [29,34,35]. In a prospective panel study from Germany, it was also observed that the introduction of flexible work schedules among employees aged 50 to 65 led to a reduction in the risk of absenteeism [36]. In contrast, in their study of male shift workers aged 50 to 59, Afsa & Giford found no significant increase in the risk of sick leave compared to individuals with fixed working hours [37]. In our sample, the vast majority of participants were female, which could explain the difference from the results of that study.

### 4.3. Association Between Ergonomics and Risk of LTSL or RWC Due to MSDs

In our sample, there was a statistically significant association between moderate or poor ergonomic work practices, as well as a lack of assistive equipment, and an increased risk of LTSL or RWC due to MSDs according to the Nurse-WIS. However, no statistically significant association was found between an increased risk of LTSL or RWC due to MSDs and the level of ergonomic knowledge. These results suggest that it is not ergonomic knowledge alone, but rather the implementation of this knowledge by nursing staff in their daily work and the existing working conditions that can lead to a reduction in the risk of LTSL or RWC due to MSDs. Thus, our findings are consistent with other publications on this topic. Two recent reviews have shown that the consistent use of ergonomic techniques in patient care reduces the physical strain on nursing staff. This leads to a reduction in the risk of MSDs and, consequently, to a reduction in the risk of absence from work due to MSDs [38,39]. A precondition for ergonomic behavior in the workplace is the sufficient availability of ergonomic aids for transferring and mobilizing patients. In a review of biomechanical studies, manual patient transfers in particular were identified as a risk factor for pain in the lumbar spine, shoulders, and knees. The authors’ recommendations included not only “no-lift” strategies but also the use of assistive devices [40]. Wiggermann et al. [12] observed that ergonomic practices in patient care are more likely to occur when sufficient assistive devices are available, when nurses are involved in planning the procurement of these devices, and when they are encouraged by their supervisors to use them. Furthermore, it was found that an adequate supply of assistive devices led to fewer nursing staff considering a job change. In their five-year follow-up study, Owen et al. [41] observed a significant decrease in MSDs, back injuries, and absence from work due to MSDs following the implementation of an ergonomic program that included assistive devices, compared to a control group. In particular, the use of ceiling and floor lifts during patient transfers had a particularly strong impact on reducing MSDs, injuries, and absenteeism [42,43,44]. Risor et al. [45], on the other hand, found no significant reduction in MSDs or absence from work due to MSDs following the acquisition of assistive devices, even though participants in the intervention group reported using the devices daily in patient care. The authors speculated that the 12-month observation period may have been too short and that positive effects on health status and workplace absenteeism might only become apparent after several years. The lack of evidence in our study for a statistically significant influence of ergonomic knowledge on the risk of LTSL or RWC due to MSDs following the Nurse-WIS confirms earlier findings that knowledge does not automatically lead to a change in behavior [46]. Several studies have observed that the regular knowledge transfer about ergonomic work practices through training sessions is not sufficient to ensure that ergonomic work practices are consistently implemented in everyday work. A combination of regular training sessions, positive changes in the working environment, and organizational support yielded the best results [47,48,49,50].

### 4.4. Strengths and Limitations

To the best of our knowledge, this study is the first to focus on identifying risk groups for LTSL or RWC due to MSDs in the nursing profession. The early identification of individuals at risk allows for timely preventive and rehabilitative measures, which could help ensure that those affected remain healthy and continue working in the nursing profession for longer. However, several limitations should be taken into account when interpreting the results. Our study is a cross-sectional survey; therefore, no conclusions regarding causal relationships can be drawn. Furthermore, the results are based on subjective reports from participants, which were provided retrospectively over a 12-month period. This may lead to recall bias among the respondents. Although participants were selected at random, given the response rate of 15.6%, selection bias cannot be ruled out. This could be attributed to the fact that individuals affected by MSDs are more likely to participate in a survey on this topic than those not affected. The non-responder analysis revealed significant differences between responders and non-responders in terms of age, gender, occupation and setting. As these characteristics may be potentially associated with the occurrence of MSDs and the risk of LTSL or RWC due to MSDs, it cannot be ruled out that the composition of the study population may have influenced the observed risk estimate. Certain groups may have been over- or underrepresented in the sample studied, meaning that the estimated risk of LTSL or RWC due to MSDs may not fully correspond to the risk of the underlying population. The results should therefore be interpreted with caution, particularly with regard to their generalizability. The lack of an effect of ergonomic knowledge could be due to the lack of variance in the responses. Since a large proportion of the respondents possessed extensive knowledge in the field of ergonomics, the values show a low degree of variation. This makes it statistically much more difficult to demonstrate significant correlations. It remains unclear to what extent this ceiling effect is attributable to the operationalization of ergonomic knowledge or to the sample’s high level of knowledge. Since the instrument is an objective knowledge test rather than a subjective assessment of ergonomic knowledge using a response scale, a ceiling effect would, to a certain extent, be inevitable if the sample possessed a high level of ergonomic knowledge. However, it is also possible that our instrument did not fully capture the sample’s total ergonomic knowledge. Furthermore, the questions relating to ergonomic knowledge and the use of ergonomic techniques in everyday practice were developed by the research team and had not undergone any prior validation. Additionally, the exclusively subjective assessment of one’s own working practices must be taken into account as a further potential cause of measurement bias. Due to a desire to conform to social norms, participants might rate their own working practices more highly than they actually are, in order to avoid any potential negative consequences of their statements. The results should therefore be interpreted with caution.

### 4.5. Future Research

Future studies should focus on geriatric care, as very few caregivers from this field participated in our survey. Furthermore, future studies should include more men, young employees, and nursing assistants in order to extend the findings to the groups that were underrepresented in our study.

In addition, future research should address not only declarative knowledge regarding ergonomic issues but also questions concerning practical competencies, the quality and frequency of the application of ergonomic techniques, and the actual workload faced by nursing staff in their daily work. Another important issue is how to address the identified risk groups within organizations in order to prevent impending LTSL or RWC due to MSDs. We are currently unaware of any publications regarding a selection of specific preventive measures that have been recommended or evaluated for such identified risk groups. Future research should address this issue.

## 5. Conclusions

Overall, the results of this study show that just over one-third of the respondents are at high risk of LTSL or RWC due to MSDs according to the Nurse-WIS. The following factors were identified as associated factors: the age of the nursing staff, shift work, a lack of assistive equipment in the facilities, and a moderate-to-poor assessment of their own ergonomic work practices. In contrast, the level of knowledge regarding ergonomic behavior in everyday nursing practice had no statistically significant association with the outcome. These results suggest that it is not ergonomic knowledge alone, but rather the application of this knowledge in everyday nursing practice and under the prevailing working conditions in the facilities that appears to be relevant for reducing the risk of LTSL or RWC due to MSDs. Ergonomic work practices reduce the physical strain on nursing staff, which could lead to a reduction in MSDs and their negative consequences for those affected, patients, and the healthcare system. A combination of behavioral and situational prevention measures is recommended, which should address both organizational and individual factors in order to support nurses’ long-term health and work ability. Furthermore, as the study design does not allow any conclusions regarding a causal relationship, future studies should be conducted using a longitudinal design.

## Figures and Tables

**Table 1:** Description of the study population (*n* = 501).

Variable	Frequency (n)/ Mean $\left(\underset{x}{¯}\right)$	Percentage (%)/ Standard Deviation (SD)
Gender (female)	432	86.2
Age (in years)		
Under 30	86	17.2
30 to 39	74	14.8
40 to 49	122	24.4
50 to 59	138	27.5
60 and older	81	16.2
BMI (kg/m^2^) (n/a = 15)		
Underweight (<18.5)	6	1.2
Normal weight (18.5–24.9)	173	34.5
Over weight (25–29.9)	148	29.5
Obesity (≥30)	159	31.7
Smoking status (yes) (n/a = 1)	127	25.4
Physical activity (in h/w) (n/a = 2)		
Less than 2	308	61.7
2 or more	191	38.3
School leaving certificate (n/a = 4)		
Lower secondary school	54	10.9
Secondary school	263	52.9
University entrance qualification	180	36.2
Occupation (n/a = 6)		
Certified health & nursing professional	296	59.8
Certified geriatric nurse	47	9.5
Nursing assistant in hospitals	40	8.1
Nursing assistant in elderly care	52	10.5
Other	60	12.1
Setting (n/a = 8)		
Hospital	284	57.6
Residential care home	25	5.1
Outpatient care	168	34.1
Other	16	3.2
Management position (yes) (n/a = 3)	97	19.5
Average working hours (in h/w) (n/a = 10)	34.7	9.9
Years in profession (n/a = 9)	20.8	13.4
Shift work (yes) (n/a = 4)	318	64.0
Employment status (n/a = 8)		
Full-time	239	48.5
Half-time	224	45.4
Other	30	6.1
Ergonomics training over the last 2 years (n/a = 2)		
Yes	335	67.1
No	164	32.9
Equipment (n/a = 5)		
Sufficient	216	43.5
Insufficient/not at all	280	56.5
Use of equipment (n/a = 39)		
Frequently	338	73.2
Moderate	101	21.9
Rarely/never	23	5.0
Ergonomic knowledge (n/a = 14)		
High	327	67.1
Low to medium	160	32.9
Ergonomic practice		
Good	239	47.7
Moderate	235	46.9
Poor	27	5.4

BMI: Body Mass Index; kg: kilogram; m^2^: square meter; h: hours; w: week; n/a: not available.

**Table 2:** High risk of LTSL or RWC due to MSDs according to the Nurse-WIS (*n* = 501).

Variable	Nurse-WIS Categories		Univariate Analysis ^a^	
	Low/ Moderate *n* (%)	High *n* (%)	OR (95% CI)	*p*-Value
Gender				
Male (ref)	49 (71.0)	20 (29.0)	1	**0.223**
Female	274 (63.4)	158 (36.6)	1.4 (0.81–2.46)	
Age (in years)				
<30 (ref)	60 (69.8)	26 (30.2)	1	**0.219**
30–39	49 (66.2)	25 (33.8)	1.2 (0.61–2.29)	
40–49	80 (65.6)	42 (34.4)	1.2 (0.67–2.19)	
50–59	78 (56.5)	60 (43.5)	1.8 (1.00–3.14)	
≥60	56 (59.1)	25 (30.9)	1.0 (0.53–1.99)	
BMI (kg/m^2^) (n/a = 15)				
<18.5	3 (50.0)	3 (50.0)	1.9 (0.39–10.13)	0.328
18.5–24.9 (ref)	115 (66.5)	58 (33.5)	1	
25.0–29.9	87 (58.8)	61 (41.2)	1.4 (0.88–2.19)	
≥30	107 (67.3)	52 (32.7)	0.9 (0.61–1.52)	
Physical activity (h/w) (n/a = 2)				
<2	190 (61.7)	118 (38.3)	1.4 (0.95–1.04)	**0.093**
≥2 (ref)	132 (69.1)	59 (30.9)	1	
Smoking status (n/a = 1)				
Yes	73 (57.5)	54 (42.5)	1.5 (0.98–2.24)	**0.060**
No (ref)	249 (66.8)	124 (33.2)	1	
School leaving certificate (n/a = 1)				
Lower secondary school	34 (63.0)	20 (37.0)	1.1 (0.61–2.04)	0.732
Secondary school (ref)	172 (65.4)	91 (34.6)	1	
University entrance qualification	115 (63.9)	65 (36.1)	1.1 (0.72–1.59)	
Other	1 (33.3)	2 (66.7)	3.8 (0.34–42.25)	
Occupation (n/a = 6)				
Health and nursing (ref)	216 (64.3)	120 (35.7)	1	0.693
Care for the elderly	61 (61.6)	38 (38.4)	1.1 (0.71–1.78)	
Other	41 (68.3)	19 (31.7)	0.8 (0.46–1.50)	
Setting (n/a = 8)				
Hospital/inpatient care (ref)	192 (62.1)	117 (37.9)	1	0.498
Outpatient care	113 (67.3)	55 (32.7)	0.8 (0.54–1.19)	
Other	11 (68.8)	5 (31.3)	0.8 (0.25–2.20)	
Employment status (n/a = 8)				
Full-time (ref)	155 (64.9)	84 (35.1)	1	0.912
Part-time	142 (63.4)	82 (36.6)	1.1 (0.73–1.56)	
Other	20 (66.7)	10 (33.3)	0.9 (0.41–2.06)	
Average working hours (h/w) (n/a = 10)				
<30 (ref)	64 (64.0)	36 (36.0)	1	0.751
30–39	131 (66.5)	66 (33.5)	0.9 (0.54–1.48)	
≥40	122 (62.9)	72 (37.1)	1.1 (0.64–1.73)	
Shift work (n/a = 4)				
Yes	195 (61.3)	123 (38.7)	1.5 (0.99–2.16)	**0.058**
No (ref)	125 (69.8)	54 (30.2)	1	
Years in profession (n/a = 9)				
<10 (ref)	93 (71.0)	38 (29.0)	1	0.263
10–19	71 (63.4)	41 (36.6)	1.4 (0.83–2.42)	
20–29	60 (60.6)	39 (39.4)	1.6 (0.92–2.76)	
≥30	91 (60.7)	59 (39.3)	1.6 (0.96–2.62)	
Management position (n/a = 3)				
Yes (ref)	63 (64.9)	34 (35.1)	1	0.910
No	258 (64.3)	143 (35.7)	1.0 (0.65–1.63)	
Ergonomic knowledge (n/a = 14)				
High (ref)	207 (63.3)	120 (36.7)	1	0.320
Low to medium	105 (65.6)	55 (34.4)	0.8 (0.55–1.21)	
Ergonomics training (n/a = 2)				
Yes (ref)	109 (66.5)	55 (33.5)	1	0.486
No	212 (63.3)	123 (36.7)	0.9 (0.59–1.29)	
Equipment (n/a = 5)				
Sufficient (ref)	157 (72.7)	59 (27.3)	1	**<0.001**
Insufficient	161 (57.5)	119 (42.5)	1.9 (1.34–2.88)	
Use of equipment (n/a = 39)				
Frequently (ref)	219 (64.8)	119 (35.2)	1	0.255
Moderate	58 (57.4)	43 (42.6)	1.4 (0.87–2.15)	
Rarely/never	17 (73.9)	6 (26.1)	0.8 (0.43–1.49)	
Ergonomic practice				
Good (ref)	176 (73.6)	63 (26.4)	1	**<0.001**
Moderate	134 (57.0)	101 (43.0)	2.1 (1.43–3.10)	
Poor	13 (48.1)	14 (51.9)	3.0 (1.34–6.75)	

^a^ Univariate logistic regression; **bold** variables were included in the multivariate model (*p* < 0.25); BMI: Body Mass Index; kg: kilogram; m^2^: square metre; h: hours; w: week; OR: Odds Ratio; 95% CI: 95% Confidence interval; Nurse-WIS: Nurse—Work Instability Scale; n/a: not available; ref: reference group.

**Table 3:** Results of the non-responder analysis (*n* = 4000).

Variable	Questionnaire Completed (*n* = 4000)		
	No (*n* = 3454) *n* (%)	Yes (*n* = 546 *) *n* (%)	*p*-Value ^a^
Gender			
female	2810 (85.6)	474 (14.4)	**0.002**
male	644 (89.9)	72 (10.1)	
Age (in years)			
<30	1210 (92.2)	103 (7.8)	**<0.001**
30–39	707 (88.8)	89 (11.2)	
40–49	564 (81.7)	126 (18.3)	
50–59	662 (79.0)	176 (21.0)	
≥60	311 (85.7)	52 (14.3)	
Occupational group			
certified nurses	2888 (85.4)	493 (14.6)	**<0.001**
care assistants	566 (91.4)	53 (8.6)	
Setting			
outpatient care	1765 (88.3)	235 (11.8)	**<0.001**
hospital	1689 (84.5)	311 (15.6)	

^a^ chi^2^-test (Pearson); * Of the respondents, 501 (91.8%) were included in the analysis; in bold: significant differences (*p* < 0.05).

## Data Availability

The data presented in this study are available on request from the corresponding author due to ethical restrictions. The informed consent obtained from the study participants did not include provisions for public deposition of the study data in a data repository or for secondary use of the data beyond the purposes specified in the consent form. Consequently, the data cannot be deposited in a public repository without exceeding the scope of the consent provided by the participants.

## Abbreviations

The following abbreviations are used in this manuscript:

BGW	German Institution for Statutory Accident Insurance and Prevention in the Health and Welfare Services
BMI	Body mass index
CI	Confidence interval
kg	Kilogram
LPEK	Local Psychological Ethics Committee at the Center for Psychosocial Medicine
LTSL	LTSL
MSDs	Musculoskeletal disorders
Nurse-WIS	Nurse—Work Instability Scale
OR	Odds ratio
RWC	Reduced work capacity
UKE	University Medical Center Hamburg Eppendorf
WI	Work instability

## Appendix A

**Table A1:** Questions on knowledge about ergonomic working.

**In the following questions, we would like to know which factors or behaviours are important for working ergonomically with people in need of care, in order to maintain the long-term health of your musculoskeletal system. Please read the questions carefully and select the answer options that you think are correct. Please note that both single and multiple answer options may be correct.**
Q 1: What should a nurse pay attention to when moving and positioning patients?
□ lift and carry with a rounded back and straight legs □ lift and carry with a straight back, bending the knees and keeping the load close to the body □ lift and carry with the load evenly distributed between both legs □ lift and carry with a straight back and arms outstretched, holding the person far from the body
Q 2: When is it advisable for nurses to attend an ergonomics training course? □ after the introduction of new work equipment and aids, to learn how to use them □ only after back pain has occurred, to prevent future episodes □ at regular intervals to refresh one’s knowledge of the techniques □ a single session is sufficient to apply the techniques in everyday practice
Q 3: What recommendations apply to desk-based work in the care sector? □ work should be carried out whilst standing □ monitor and chair should be adjusted to correct height □ posture at the desk is not important □ sitting position should be changed more frequently
Q 4: Which of the following measures help to reduce ergonomic strain on the nurse when assisting a patient with personal hygiene? □ adjusting the bed to hip height □ leaning forward significantly to improve access to the person being cared for □ nurse occasionally uses a stool themselves when washing a person in the bathroom □ carrying out all tasks whilst standing to ensure a better overview
Q 5: Which aids can be used to reduce strain during patient transfers? □ sliding mats □ lifts □ non-slip shoes □ sliding boards
